# Nonoperative management of high degree hepatic trauma in the patient with risk factors for failure: have we gone too far?

**Published:** 2010-08-25

**Authors:** M Beuran, I Negoi, AT Ispas, S Păun, A Runcanu, G Lupu, D Verter

**Affiliations:** * >Emergency Hospital of BucharestRomania; ** >Anatomy Department, ‘Carol Davila’ University of Medicine and Pharmacy, BucharestRomania

**Keywords:** hepatic trauma, nonoperative management, risk of failure

## Abstract

**Background**: Nonoperative management (NOM) of liver trauma is currently rather the rule than the exception. However, the current evidence presents subgroups of patients at higher risk for NOM failure. These patients must be treated more cautiously regarding the NOM approach.

**Method**: A case report of 3 polytrauma patients (Injury Severity Score>17) with high–degree liver trauma managed nonoperatively.

**Results**: The first case presented is the one of a polytrauma patient with degree Ⅳ liver injury and impaired mental status. It was a high risk for NOM failure because there was an angiographically hemostasis. The second case is one of a polytrauma patient who became hemodynamically stable after the administration of 2000 ml of fluid intravenously. There was a nonoperative approach with angiography and embolization of degree Ⅳ liver injury. Despite the success of the nonoperative treatment, there was an important hepatic necrosis following embolization. The third case is one of a polytrauma patient with a degree Ⅳ hepatic injury. Success was accomplished in NOM without an angiography.

**Conclusions**: Nonoperative management of liver injuries can be applied safely even in high degree hepatic trauma. In hemodynamically metastable patients or impaired mental status patients, the nonoperative approach can be applied successfully, but the trauma surgeon must be very cautious.

Introduction: The prevalence of the blunt liver trauma has increased over the past 3 decades [1]. It is not known whether this is an absolute increase in liver injuries or a false increase due to a better Computed Tomography (CT) diagnosis and the existence of trauma registries [1]. 

The liver, the most voluminous solid organ of the abdomen is frequently injured by both, blunt and penetrating abdominal trauma. Although in the urban setting the penetrating abdominal wounds may be more common, about 85% of liver lesions are produced by blunt trauma [2]. The required velocity to damage the liver generates associated visceral injuries in 65% of the penetrating trauma and in 10% of the blunt trauma [2]. 

Over the past 20 years, there have been fundamental changes in the liver trauma approach. There was a major role in the recognition that most liver injuries stop bleeding spontaneously [3–5]. Moreover, the abdominal CT is available in more and more trauma centers and it is the most valuable method used in the evaluation of the blunt trauma, in hemodynamically stable patients [6]. As a consequence, the nonoperative management (NOM) has become the standard therapy for over 80% of blunt liver trauma, with a success rate exceeding 95% [5;7]. The main condition for starting the NOM is hemodynamic stability [8;9]. The peritoneal signs and/or hemodynamic instability are absolute indications to perform a laparotomy [10]. The current evidence indicates that the NOM can be successfully applied to selected patients who are initially hemodynamically unstable but respond to intravenous fluids [11]. Over the past years the operative management of liver trauma has changed, with an increasingly use of packing techniques, damage control and early angiography with embolization of surgical approached lesion [7]. 

Case 1: A 14–year–old male patient was admitted for a traffic related accident. The patient was mechanically ventilated through an orotracheal tube at the accident site. 

 In the emergency room  the blood pressure (BP) was of 90/65 mm Hg, the heart rate (HR)=120 bpm, and the oxygen saturation (SpO2) 97%. On clinical examination the patient had two traumatic marks at the head (Abbreviated Injury Scale – AIS Head= 3, AIS Face= 2), as well as thoracic and abdominal trauma. The hemodynamic stability was maintained by intravenous crystalloids administration. 

**FAST ultrasound (Focused Abdominal Sonography for Trauma)** made by the imagist physician showed perihepatic fluid. **Thoracic Computed Tomography (CT)** showed right hemothorax (Organ Injury Scale – OIS= Ⅰ, AIS= 2) and bilateral lung basal contusions (OIS= Ⅱ, AIS= 3) (Figure 1).

**Figure 1:**
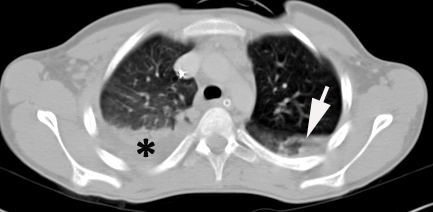
Thoracic CT. Arrow – lung contusion. Hemothorax

Abdominal CT showed degree Ⅳ (OIS) liver laceration, AIS= 4, at the level of the Ⅶ and the Ⅷ Couinaud segments, with minimum hemoperitoneum (Figure 2, Figure 3). 

**Figure 2:**
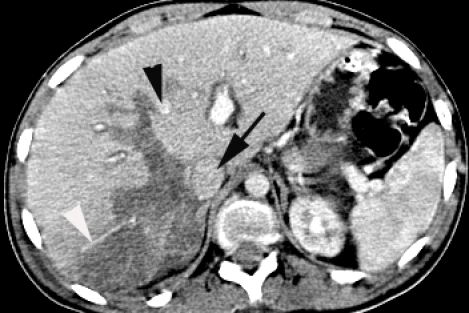
Abdominal CT. Degree Ⅳ liver laceration. White arrowhead – branch of right hepatic vein. Black arrow  – inferior vena cava. Black arrowhead – middle hepatic vein

**Figure 3:**
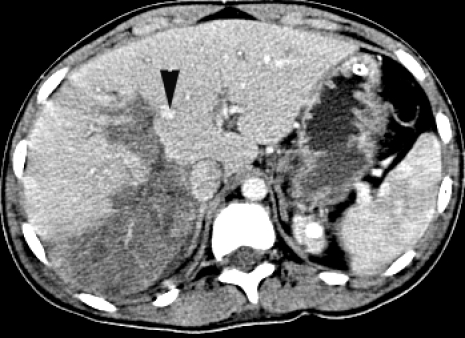
Abdominal CT. Degree Ⅳ liver laceration. Black arrowhead – middle hepatic vein.

Although the patient was tracheally intubated and mechanically ventilated, being intravenously sedated, the nonoperative management of the liver injury was decided due to the patient's hemodynamic stability.

Due to the high degree of the liver injury, it was decided that an angiography should be carried out. The active bleeding detected in the right hemiliver was embolized (Figure 4).

**Figure 4:**
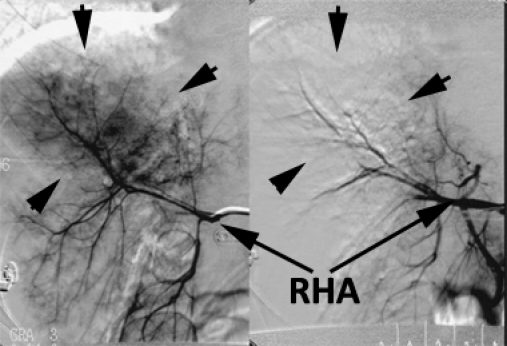
Angiography before and after embolization of the bleeding source from the right hemiliver (arrows). RHA – right hepatic artery.

The post–embolization clinical course was favorable, the patient being discharged 20 days after admission.

 Case 2: A 28–year–old male patient was admitted after a workplace accidental explosion. During the primary survey, the patient was fully conscious, with Glasgow Coma Scale (GCS) = 15, BP = 80/55 mm Hg, HR = 115 bpm and respiratory rate (RR) = 27/min, SpO2 = 96%.On the clinical examination the patient experienced a minor craniocerebral trauma, a facial trauma with open wounds at this level (AIS = 2), thoracic trauma with penetrating wounds at the level of the right thorax, produced by metallic fragments, multiple wounds in the left upper limb caused by metallic fragments (AIS = 3).In the emergency room, the patient received resuscitating crystalloids intravenous fluids. During clinical and imagistic examination, the patient received 2000 ml of crystalloids solutions having a secondary blood pressure rate of 110/60 mm Hg.

**The FAST ultrasound** made by the imagist physician did not show free intraperitoneal fluid. **The Chest X–Ray** showed right pneumothorax with metallic fragments in the right thorax and in the right superior abdominal quadrant (Figure 5).

**Figure 5:**
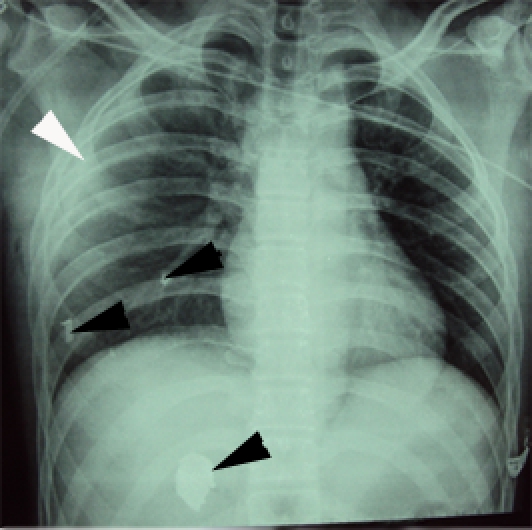
Chest X–Ray. White arrow – pneumothorax. Black arrows – metallic fragments (right thorax and right upper abdominal quadrant).

**Cerebral CT** showed no intracranial lesions. However, there was a contusion of the right facial soft tissues.

**Thoracic CT**: metallic fragments in the adipose tissue of the right thorax, corresponding to the Ⅴ–Ⅷ ribs and a metallic fragment at the level of the middle lobe of the right lung (Figure 6).

**Figure 6:**
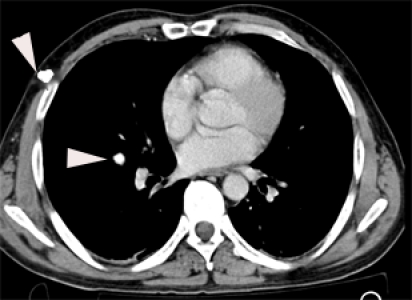
Thoracic CT. White arrows – metallic fragments (subcutaneous and middle lobe of the right lung).

**Abdominal CT**: degree Ⅳ (OIS) Iiver laceration, AIS = 4, at the level of the right hemiliver, Ⅴ, Ⅶ, Ⅷ Couinaud segments (Figure 7). We could see 2 metallic fragments at the level of the Ⅴ and Ⅷ Couinaud segments (Figure 8, Figure 9).

**Figure 7:**
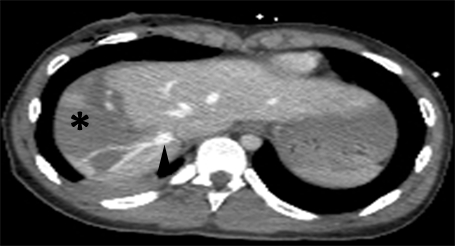
Abdominal CT. Degree Ⅳ liver laceration. Black arrow – right hepatic vein.

**Figure 8:**
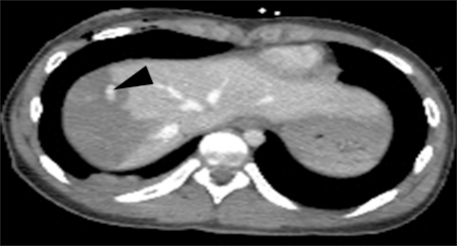
Abdominal CT. Degree Ⅳ liver laceration. Black arrow – segment Ⅷ metallic fragment

**Figure 9:**
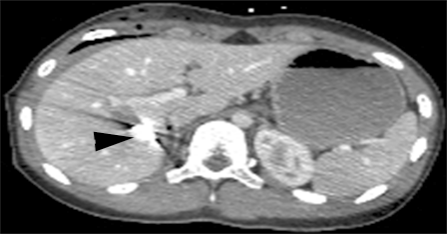
Abdominal CT. Black arrow – segment Ⅴ metallic fragment.

At the level of the Ⅴ Couinaud segment, there was an important arterial–venous fistula (Figure 10). The fistula was made by a branch of the replacing right hepatic artery (branching off the superior mesenteric artery) and a portal branch (Figure 11). The patient also presented, a Ⅱ degree (OIS) right kidney laceration (AIS = 2).

**Figure 10:**
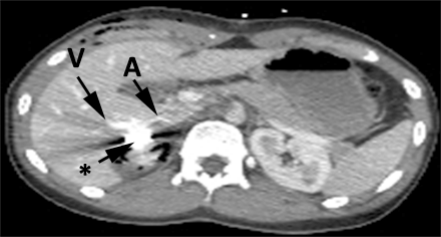
Abdominal CT. Black arrow –metallic fragment. A – replacing right hepatic artery branch (branching off the superior mesenteric artery). Ⅴ – portal branch. Arteriovenous fistula compatible aspect.

**Figure 11:**
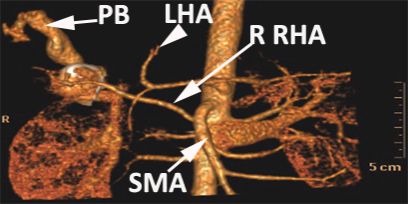
CT angiography. PB – portal branch. SMA – superior mesenteric artery. R RHA – replacing right hepatic artery. LHA – left hepatic artery.

Because of his hemodynamic stability after fluids infusion (hemodynamically metastable patient), it was decided that a nonoperative management of the liver injury with early angiographic embolization of arterio–venous fistula (Figure 12, Figure 13, Figure 14) should be done. The upper limb, facial and right thorax wounds were surgically approached, cleaned and primarily sutured. 

**Figure 12:**
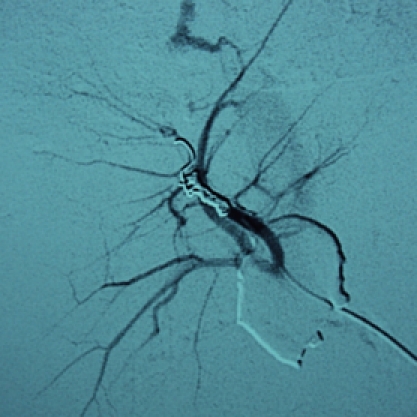
Angiography with contrast in the right hepatic artery. We can observe arterial – portal fistula.

**Figure 13:**
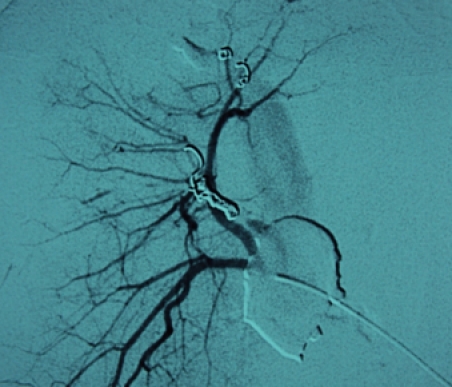
Angiography with contrast in the right hepatic artery, after arteriovenous fistula embolization.

**Figure 14:**
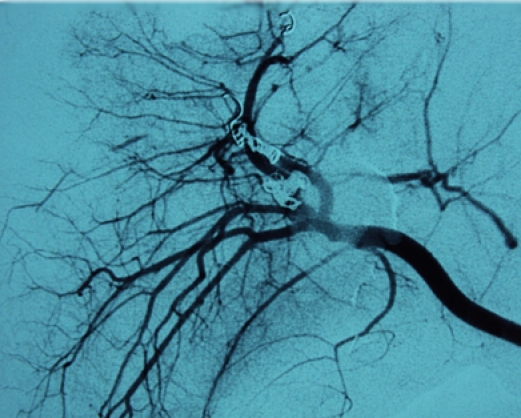
Angiography with contrast in the right hepatic artery, after all bleeding sources embolization.

In the post–embolization period, the patient experienced an important hepatic necrosis: AST = 1720 UI/L, ALT = 1927 UI/L, Total Bilirubin = 6.20 mg/d (Figure 15). On clinical examination, the patient complained of right upper abdominal quadrant pain associated with fever between days 5–8.

**Figure 15:**
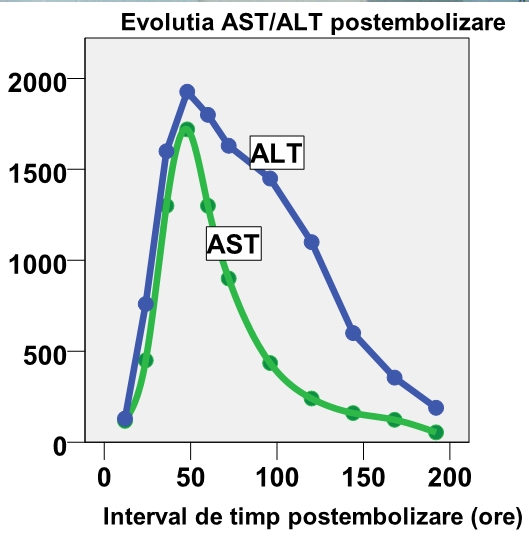
AST/ALT post embolization evolution

The 10 days post embolizaton follow-up abdominal CT showed a favorable development of the liver laceration area (Figure 16). Due to his favorable clinical course, the patient was discharged 2 weeks after admission. 

**Figure 16:**
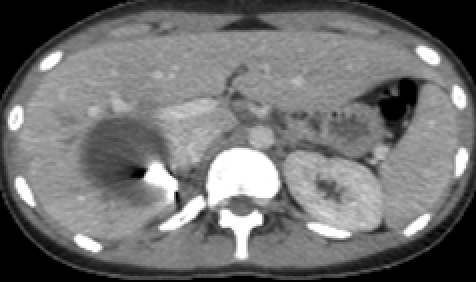
Follow–up CT scan, 10 days after embolization

The follow–up CT scan performed 6 weeks later showed a favorable development of the liver lesion (Figure 17). 

**Figure 17:**
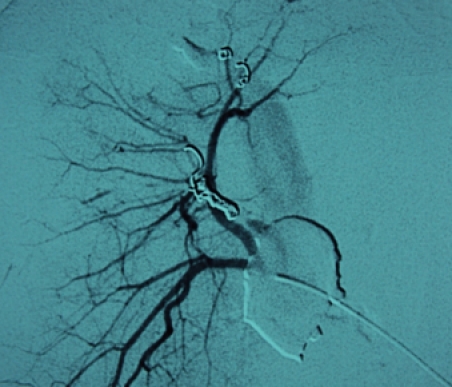
Follow–up CT scan, 6 weeks after embolization.

Case 3: A 38–year–old patient was admitted after an accidental fall. In the emergency room, the patient experienced right thoracic and right upper quadrant abdominal pain. His BP was of 110/70 mm Hg, HR = 95 bpm, RR = 32/min, SpO2 = 95 %, GCS = 15.

**FAST ultrasound** made by the imagist physician showed an average amount of free intraperitoneal fluid (perihepatic, Douglas and between intestinal loops) in the right pleural cavity. **Chest X–Ray** showed right hemothorax with pneumothorax, multiple rib fractures and right clavicle fracture.**Thoracic CT scan**: right pneumothorax with mass effect on mediastinal structures, right hemothorax (OIS Thoracic Vascular = Ⅰ, AIS = 2), upper lobe laceration of the right lung, bilateral basal pulmonary contusions (OIS Lung = Ⅲ, AIS = 4), C Ⅱ – C Ⅶ unilateral flail chest, C Ⅱ – C X rib fractures (OIS Thoracic Wall – Ⅳ, AIS = 4) (Figure 18).

**Figure 18:**
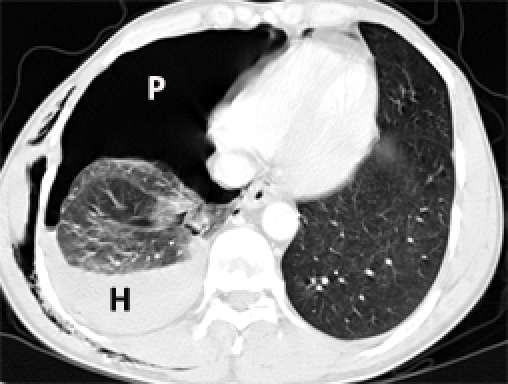
Thoracic CT. P – pneumothorax. H – hemothorax

**Abdominal CT**: Contusion of the Ⅵ, Ⅶ and Ⅷ liver segments, with a deep liver laceration adjacent to the hilum area (Figure 19, Figure 20). Moreover, a moderate amount of free intraperitoneal fluid was seen in the Morison area and the perihepatic and paracolic gutters.

**Figure 19:**
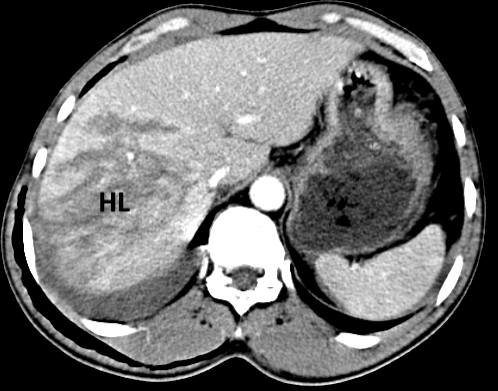
Abdominal CT. Degree Ⅳ liver laceration.

**Figure 20:**
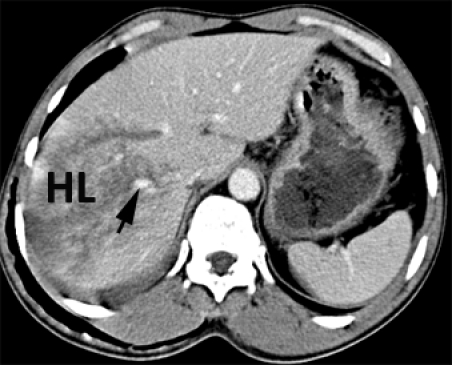
Abdominal CT. Degree Ⅳ liver laceration. Arrow – right hepatic vein.

A thoracic tube for drainage was inserted. Due to the patient's hemodynamic stability, it was decided that a nonoperative approach of the liver trauma should be done. The patient was discharged after 9 days

Discussion: Although the spleen and the liver are the most commonly injured organs in blunt abdominal trauma, the large use of the CT revealed that the liver and not the spleen is the most frequently injured organ in the body [12]. The current evidence indicates that the most common liver injuries that can be managed nonoperatively are degree Ⅰ – Ⅲ and up to 60% of d Ⅳ – Ⅵ degree [8]. 

For a safe application of the selective nonoperative management in the liver trauma, the following parameters have to be respected: (1) hemodynamic stability or patients who become stable after a moderate amount of fluid replacement, (2) accurate CT characterization of the liver lesions and a small amount of free intraperioneal fluid, (3) the absence of other abdominal lesions requiring laparotomy, (4) < 4 units of transfused blood [13]. 

One of the major fears of trauma surgeons dealing with nonoperative management is the possibility to initially miss an injury. Miller et al. showed that the frequency of associated abdominal injuries is of 5% in liver trauma and of 1,7% in spleen trauma [14]. The associated intestinal (11% vs. 0%, p =. 0004) and pancreatic (7% vs. 0%, p =. 007) lesions were more common in patients with liver compared to spleen trauma. Liver trauma patients managed nonoperatively had in 2.3% of the cases an initially undiagnosed abdominal injury. This study concludes that the rate of associated missed injuries is low and should not influence the nonoperative management of hepatic lesions [14]. 

Initially, it was thought that the patients with an impaired mental status are not suitable candidates for nonoperative management, but today, it has been proven that the failure rate of nonoperative approach is not higher in this subgroup of patients [15;16]. 

As the trauma surgeons became familiar with the nonoperative therapy, the hepatic injuries of increased gravity were approached nonoperatively. High degree liver lesions, with a large amount of hemoperitoneum can be managed nonoperatively if the patient is hemodynamically stable [17]. However, these patients should be closely followed by a trained nursing staff, able to early recognize the peritoneal signs and ongoing hemorrhage. 

Although traditionally the patients with blunt solid abdominal organs trauma were restricted to bed rest and light physical activity, there is no data to support this recommendation. The timing of mobilization of patients with blunt solid organ injuries does not seem to contribute to delayed hemorrhage requiring laparotomy [18]. 

The current medical literature shows that degree Ⅲ or lower liver injuries in hemodinamically stable patients should not repeat computed tomographic scan before discharge, assuming the low rate of failure in these patients [19;20]. In contrast, because the prevalence of complications is higher for more severe injuries (degree Ⅳ or Ⅴ), follow–up CT may be necessary in this subset of patients to identify potential complications that are amenable to early interventions. The optimal time frame for follow–up CT in patients with high–degree injuries appears to be between 7 and 10 days from the original injury [21]. 

The complications of liver lesions nonoperatively treated are hemobilia (0.2 – 0.3%), delayed hemorrhage (<3%), liver abscess and biloma (< 0.5%) and extrahepatic bile duct injury [2]. These complications are more frequent in high degree liver trauma, but fortunately, most can be addressed by minimally invasive techniques [13;22].

The process of hepatic repair after blunt trauma follows a predictable pattern: hemoperitoneum usually resolves within 1 week, subcapsular hematomas in 6 – 8 weeks and liver laceration in 3 weeks [21]. Hepatic hematomas and bilomas may persist for years. Parenchymal homogeneity is restored in 4 – 8 weeks [21].

By increasing the frequency of selective nonoperative management use in abdominal trauma, another important issue emerges: the education of the residents in abdominal emergency surgery. The use of modern imagistic tools decreased the nontherapeutic laparotomy rate as a result of positive diagnostic peritoneal lavages from 35% to 14%. However, as the nonoperative experience grows, the resident's opportunity for operative experience decreases [1;23]. 

Lucas and Ledgerwood highlight the inherent challenges of achieving psychomotor skills for the hemostasis of solid organ injury in an era of nonoperative therapy [24]. This study reviews all patients with liver injury seen during 24 months in five consecutive decades with a special attention to the number of surgeries performed by a resident during training [24]. Initially (1960s), all the injuries were explored, nowadays (2000s) most injuries are observed. The number of patients was of 235 (1960s), 228 (1970s), 79 (1980s), 116 (1990s) and 64 (2000s). The highest number in the 1990s reflects the diagnosis of minor, clinically insignificant, blunt injuries after the CT became available. During training, a resident performed an average of 12, 12, 2.4, 4 and 1.3 procedures for hemostasis. The authors conclude that the residents will need to supplement their clinical experience with solid organ hemostasis by practicing on appropriate animal models and cadaver dissections [24]. 

Conclusions: Selective nonoperative management of blunt hepatic lesions is the treatment modality of choice in hemodinamically stable patients, irrespective of the degree of injury. It is associated with a low overall morbidity and mortality and the result does not increase the length of stay, need for blood transfusions and bleeding complications compared with operative management. In polytrauma setting, the nonoperative management of liver injuries can be challenging. However, it can be quite satisfying to be able to successfully manage patients with severe and multiple traumatic injuries in nonoperative fashion. Modern imagistic tools can accurately depict various patterns of hepatic parenchymal injuries as well as associated bowel or pancreatic injuries that require an emergency laparotomy. Reduced incidence and decreased therapeutic laparotomies for liver injury have created a training vacuum for future trauma surgeons. 
